# Development of the Global Mycetoma Working Group

**DOI:** 10.1093/trstmh/traa163

**Published:** 2021-04-14

**Authors:** Rita M. Traxler, Karlyn D. Beer, David D. Blaney, Wendy W. J. van de Sande, Ahmed H. Fahal, Kingsley B. Asiedu, William A. Bower, Tom Chiller

**Affiliations:** a National Center for Emerging and Zoonotic Infectious Diseases, Centers for Disease Control and Prevention, 1600 Clifton Road NE MS 24-12, Atlanta GA 30329, USA;; b Department of Medical Microbiology & Infectious Diseases, Erasmus Medical Center, Na-903, Dr. Molewaterplein 40, 3015 GD Rotterdam, The Netherlands;; c Mycetoma Research Center, Soba University Hospital, PO Box 102, Khartoum, Sudan;; d Department of Control of Neglected Tropical Diseases, World Health Organization, WHO Headquarters, Avenue Appia 20, 1211, Geneva 27, Switzerland

**Keywords:** coalition, global health, mycetoma, neglected tropical disease

## Abstract

The Global Mycetoma Working Group (GMWG) was formed in January 2018 in response to the declaration of mycetoma as a neglected tropical disease (NTD) by the World Health Assembly. The aim of the working group is to connect experts and public health practitioners around the world to accelerate mycetoma prevention activities and reduce the impact of mycetoma on patients, healthcare providers and society in the endemic regions. The working group has made tangible contributions to mycetoma programming, awareness and coordination among scientists, clinicians and public health professionals. The group’s connectivity has enabled rapid response and review of NTD documents in development, has created a network of public health professionals to provide regional mycetoma expertise and has enabled mycetoma to be represented within broader NTD organizations. The GMWG will continue to serve as a hub for networking and building collaborations for the advancement of mycetoma clinical management and treatment, research and public health programming.

## Need and vision of a global working group

Mycetoma is a chronic and progressive infection caused by a variety of environmental fungi (eumycetoma) and bacteria (actinomycetoma). As a neglected tropical disease (NTD) of the skin, mycetoma primarily affects the feet and hands, causing disability and stigma that often reinforces cycles of poverty among low-income equatorial populations who are at greatest risk.^1^ Mycetoma was recognized as an NTD at the 69th World Health Assembly (WHA) in May 2016.^2^ The WHA resolution set forth goals to reduce the impact of mycetoma and the public health and socio-economic burdens that the disease places on poor, rural communities worldwide. This WHA resolution spurred conversations centred on the need to improve global coordination of mycetoma-related activities. The value of coordination through a Global Mycetoma Working Group (GMWG) became more apparent as participants exchanged strategies and information during the 24 May 2017 informal consultation on mycetoma sponsored by the World Health Organization (WHO) in Geneva, Switzerland. Specifically, the role of the working group would be coordination of public health advocacy and strategic planning for a measurable reduction in mycetoma burden, a mechanism to measure such progress and assisting in the integration of mycetoma programming within the larger global NTD community.

The GMWG was conceived as an international consortium to connect mycetoma researchers, clinicians and regional mycetoma working groups, as well as established NTD programs.^3^ The aim of the group is not to duplicate, displace or supplant the activities of research-oriented groups, such as the International Society for Human and Animal Mycology (ISHAM) Working Group on eumycetoma. Instead, the goal of the GMWG is to engage in activities that support, connect and amplify ongoing research to strengthen mycetoma prevention and treatment. The objectives of the GMWG are to raise awareness of mycetoma; develop the programs, strategy and funding to address the WHA resolution to reduce the burden of mycetoma and pursue control and prevention activities.

## Establishing the GMWG

Mycetoma experts around the globe were identified and invited to the GMWG by attendees of the WHO Geneva Skin NTD meeting in March 2017. The inaugural meeting of the GMWG was held on 11 January 2018 and was attended by >35 individuals from 16 countries on six continents. The meeting brought together experts on both eumycetoma and actinomycetoma from a wide range of fields, including clinical research, diagnostic tool development, drug discovery/pharmaceuticals, epidemiology, ethics, laboratory research, medical practice, health policy and program planning and development. At this inaugural gathering, attendees and group participants such as academic institutions, non-governmental organizations (NGOs), governmental organizations (e.g. Centers for Disease Control) and international organizations (e.g. WHO) were represented.

**Figure 1.**
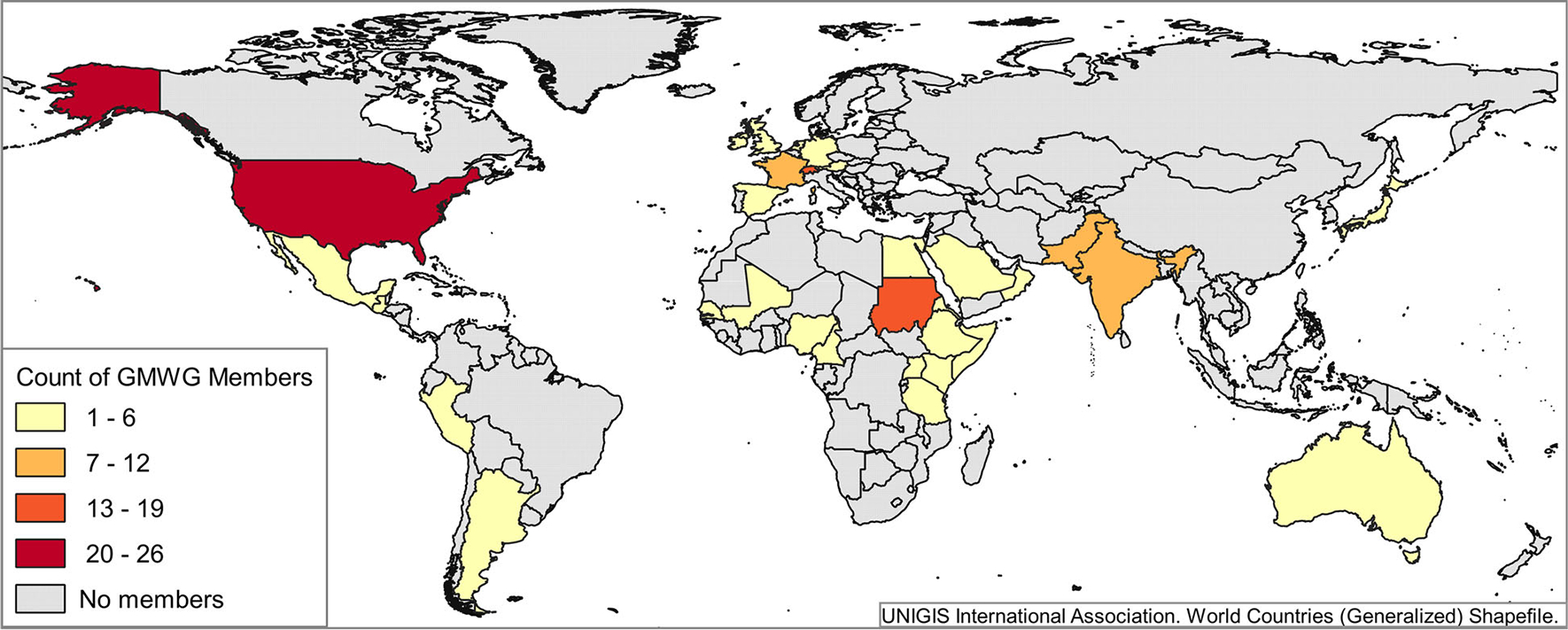
Countries of residence of GMWG members.

The GMWG is led by a steering group with representation from several different countries and continents. Working group membership has expanded to 135 individuals from 28 countries (as of 16 June 2020) (Figure 1); growth has come from word of mouth, communication with NTD stakeholders and hosting mycetoma events at various NTD and mycology conferences. Participant backgrounds remain as broad and varied as among participants at the inaugural meeting.

We identified several operational considerations for establishing and facilitating the development of the working group, particularly to make the group useful to its members. These included a quarterly virtual meeting, an online group collaboration platform, an outward-facing website and a quarterly electronic newsletter; the latter two are still under development. In addition, several priorities were identified, including epidemiology, surveillance, mapping and burden estimates; case management capacity in endemic areas; prevention, awareness and communication strategy; and monitoring, intervention evaluation and research (activities are described in Table 1). To facilitate concrete action toward these priorities, the working group elected to divide itself into topic-specific subgroups composed of members with common interests and expertise. For example, the clinical care and treatment subgroup is developing a clinical guidance document, while the epidemiology and burden estimation subgroup is focused on assembling available surveillance and clinical data in order to publish an updated burden estimate. The latter subgroup is advocating that countries publish or share their surveillance data to more accurately estimate the global burden of mycetoma, such as recent publications from Senegal and Uganda.^4,5^

## Contributions of the GMWG

Without dedicated or long-term funding, the GMWG has made tangible contributions to mycetoma programming, awareness and coordination among researchers, clinicians and public health professionals. For example, although mycetoma is a recent addition to the WHO list of NTDs, the GMWG has facilitated mycetoma representation within long-standing communities of practice focusing broadly on skin NTDs. The NTD NGO Network (NNN) is a large global community of NGOs that provides a forum for partners working to improve the health of populations experiencing poverty who are also at risk of NTDs. The NNN has a cross-cutting group dedicated to skin-manifesting NTDs, such as leprosy, scabies, podoconiosis and yaws. Through the GWMG, public health professionals advocating for mycetoma were introduced to the NNN skin NTD group and are now regularly attending members. Involvement in these groups has raised awareness of mycetoma among skin NTD professionals, some of whom have joined the GMWG, and these connections may help develop more mycetoma expertise in the NTD community.

The GMWG also provides an efficient and comprehensive resource of subject matter experts for the WHO and global public health leadership looking for mycetoma expertise. GMWG members provided important technical input and contributed written materials that were incorporated into the WHO’s 2030 NTD Roadmap, which was endorsed by the WHA.^6^ Utilizing the working group’s connectivity, soliciting the needed expertise was rapid and efficient, enabling the WHO to quickly identify relevant experts from a larger group of professionals than would otherwise be possible through a more decentralized solicitation (e.g. individual e-mails to known colleagues).

Finally, membership in global working groups like the GWMG can provide essential resources to members. With limited to no funding, powerful web-based collaboration platforms (e.g. Slack) can be leveraged to facilitate and organize communication and to share important resources that may not otherwise be available to all members. The GMWG online collaboration platform has become a forum to match senior investigators and clinicians with interested student groups around the world and can be used to seek advice from mycetoma experts on difficult cases. In addition, GMWG uses an informal e-mail list with >130 members, both of which have enabled members to share information ranging from funding proposal templates and opportunities to ongoing research activities and recognition of significant milestones and publications. While it is impossible to quantify the exact public health impact of an expanded and strengthened network of professionals dedicated to reducing the burden of mycetoma, the GMWG has enabled new interactions and collaborations that may not otherwise have been possible.

## Challenges

Although a global working group dedicated to a singular effort can be impactful, creating and maintaining such a group is time and resource intensive, and maximizing impact requires organizational leadership with sufficient access to both time and resources. While the GWMG is a coalition of professionals whose work encompasses mycetoma, GMWG participation is voluntary and ancillary to members’ primary work. Although the group enables collaboration and synergy that can enhance and complement individual members’ progress toward important disease prevention milestones, maintaining the momentum and time commitment required to complete GWMG goals and deliverables has been a challenge.

One way to maintain momentum within a volunteer coalition with limited resources is a dedicated coordinator. Creating such a role requires some committed funding but can maintain and build the coalition, expand external partnerships, identify funding for support and expansion of mycetoma activities and motivate members to reach GMWG goals. With the addition of a dedicated coordinator to focus exclusively on mycetoma-related coalition work, the GWMG would be poised to make greater global contributions to mycetoma burden estimation, clinical care and prevention, among others. Other NTD coalitions such as Footwork, an organization dedicated to podoconiosis prevention, have achieved important milestones when they have been able to retain a dedicated coordinator.

Finally, with a membership of >130 professionals from >20 countries, the ability to quickly communicate, share and store information internally and externally is critical. In its short existence, the GWMG has been and still is experimenting with novel ways to establish and allow this kind of advanced collaboration. The GWMG has evolved from using a basic e-mail list to a free account with a leading online collaboration tool, which streamlines internal mycetoma-related communication. An outwardfacing website is a key component that, because of limited resources, has not been achieved. Such a web presence could expand the reach and impact of the group by connecting members to other public health and clinical organizations with similar goals.

The GMWG, like other public health coalitions, has faced many challenges and has yet to realize its maximum potential. These challenges have attenuated the impact of the GWMG, although the group continues to make progress in spite of them.

## Future directions and how to join

Although the GMWG’s footprint remains small, there is excitement within its membership to contribute to reducing the impact of mycetoma. Areas of specific interest and expertise within the group include epidemiology and burden estimation; basic science of the host and pathogen; clinical care; diagnostics and treatment; and mycetoma programming, partnerships and community engagement. The working group continues to welcome new members and build relationships across the globe, build collaborations and produce funding proposals in order to drive the key activities needed to reduce the burden of this disease. Future workshops will be held to target specific priority tasks or research questions, participate in broad NTD activities and contribute to NTD policy development. These activities will also aim to identify and engage professionals in countries where there is little mycetoma engagement, to develop and expand collaborations to achieve GMWG priorities.

The structure may continue to evolve to accommodate growing membership, developing partnerships and requests for funding or review, however, the objectives will remain to raise awareness of mycetoma; develop the programs, strategy, and funding to address the WHA resolution to reduce the burden of mycetoma; and pursue control and prevention activities.

To join, complete the GMWG survey at https://tinyurl.com/y85mmx8v.

## Figures and Tables

**Table 1.** Identified priorities for mycetoma research, clinical practice and community action

Priorities for research	Priorities for clinical practice and community action
Update the global burden estimate and map cases	Develop a clinical resource guide for clinicians treating mycetoma cases
Develop novel point-of-care diagnostics for low-resource settings	
	Engage NTD groups to include mycetoma in new and existing prevention and awareness programs
Develop novel treatment solutions, including drug discovery and susceptibility testing	
Ascertain the mode of transmission	
Determine the role of the host and the pathogen in mycetoma	

## Data Availability

The aggregated data used to produce the figure are available from the corresponding author upon reasonable request.
